# Development and Implementation of an eHealth Oncohematonootric Program: Descriptive, Observational, Prospective Cohort Pilot Study

**DOI:** 10.2196/49574

**Published:** 2024-04-08

**Authors:** Beatriz Sánchez-Quiñones, Cristina Antón-Maldonado, Nataly Ibarra Vega, Isabel Martorell Mariné, Amparo Santamaria

**Affiliations:** 1 Hybrid Hematology Department University Hospital Vinalopó Alicante, Elche Spain; 2 Hematoinnova Unit Fundación para el Fomento de la Investigación Sanitaria y Biomédica de la Comunidad Valenciana Valencia Spain; 3 Nutrition Department Nootric Inc Barcelona Spain

**Keywords:** Nootric app, oncohematology patient, physical-nutritional well-being, multidisciplinary team

## Abstract

**Background:**

In oncohematology, both the development of the disease and the side effects of antineoplastic treatment often take a toll on patients’ physical and nutritional well-being. In this era of digital transformation, we launched a pioneering project for oncohematologic patients to promote adherence to a healthy lifestyle and improve their physical and nutritional well-being. We aim to achieve this goal by involving doctors and nutritionists through the Nootric app.

**Objective:**

This study aims to assess the impact of the use of eHealth tools to facilitate nutrition and well-being in oncohematologic patients. We also aim to determine the usefulness of physical-nutritional management in improving tolerance to chemotherapy treatments within routine clinical practice.

**Methods:**

We designed a descriptive, observational, longitudinal, prospective cohort pilot study that included a total of 22 patients from March to May 2022 in the Vinalopó University Hospital. The inclusion criteria were adults over 18 years of age diagnosed with oncohematological pathology in active chemotherapy treatment. An action plan was created to generate alerts between the doctor and the nutritionist. In the beginning, the patients were trained to use the app and received education highlighting the importance of nutrition and physical exercise. Sociodemographic, clinical-biological-analytical (eg, malnutrition index), health care impact, usability, and patient adherence data were collected. Tolerance to chemotherapy treatment and its health care impact were evaluated.

**Results:**

We included 22 patients, 11 (50%) female and 11 (50%) male, ranging between 42 and 84 years of age. Among them, 13 (59%) were adherents to the program. The most frequent diseases were lymphoproliferative syndromes (13/22, 59%) and multiple myeloma (4/22, 18%). Moreover, 15 (68%) out of 22 patients received immunochemotherapy, while 7 (32%) out of 22 patients received biological treatment. No worsening of clinical-biological parameters was observed. Excluding dropouts and abandonments (n=9/22, 41%), the adherence rate was 81%, established by calculating the arithmetic mean of the adherence rates of 13 patients. No admission was observed due to gastrointestinal toxicity or discontinuation of treatment related to alterations in physical and nutritional well-being. In addition, only 5.5% of unscheduled consultations were increased due to incidents in well-being, mostly telematic (n=6/103 consultation are unscheduled). Additionally, 92% of patients reported an improvement in their nutritional habits (n=12/13), and up to 45% required adjustment of medical supportive treatment (n=5/11). There were no cases of grade 3 or greater gastrointestinal toxicity. All of this reflects improved tolerance to treatments. Patients reported a satisfaction score of 4.3 out of 5, while professionals rated their satisfaction at 4.8 out of 5.

**Conclusions:**

We demonstrated the usefulness of integrating new technologies through a multidisciplinary approach. The Nootric app facilitated collaboration among the medical team, nutritionists, and patients. It enabled us to detect health issues related to physical-nutritional well-being, anticipate major complications, and mitigate potentially avoidable risks. Consequently, there was a decrease in unscheduled visits and admissions related to this condition.

## Introduction

### Background

Hematological malignancies encompass a heterogeneous group of diseases that have different behaviors, evolution, treatments, and prognoses. However, all of them similarly compromise the patient’s nutritional and physical status. This is because both the development of the disease and antineoplastic treatment can lead to caloric-protein malnutrition, leading to a high prevalence of adverse effects in daily clinical practice [1]. New treatments and conventional chemotherapy lead to toxicity in the gastrointestinal tract, which has a direct impact on the patient's well-being and survival [2-9]. Medical management is often insufficient to carry out a comprehensive assessment of the patient’s physical and nutritional well-being. Therefore, professional support in this aspect through the application of information and communication technologies (ICTs) in patients’ everyday environment outside the hospital is a useful tool to improve well-being and reduce health care costs [6,10-12]. There is increasing evidence showing that lifestyle interventions can improve symptoms, quality of life, and even overall survival rates for patients with cancer. Digital interventions can help implement physical-nutritional behavior modifications and empower patients through healthy lifestyle education and support [13].

An effective system for patient physical-nutritional monitoring and treatment after nutritional risk assessment appears to be lacking. We identified the need for a standardized system to prevent and treat malnutrition related to these diseases. Currently, there are studies involving mobile apps for nutritional control and support to monitor dietary intake among patients who are hospitalized and face nutritional risks. These apps have demonstrated good acceptance among patients and have the potential to be useful dietary evaluation tools for use in clinical practice. These results suggest that such tools could be extrapolated to the field of oncohematology consultations [14].

The studies available so far confirm that the application of mobile apps, among other appropriately designed digital interventions, can be effective tools in nutritional interventions [2,3,15]. It is estimated that 59% or more of the currently available apps are health-related [2]. Some studies show that mobile app interventions can improve the quality of life of patients with malignant hemopathies by reducing symptoms [16].

However, many of these apps are not developed by nutrition experts, validated by official agencies, or part of routine use in the hospital setting. The use of mobile apps for nutritional interventions to improve dietary patterns, avoid or reduce side effects, and improve patient physical and nutritional well-being is a new challenge currently facing health care.

Therefore, we have launched a pioneering project with the aim of improving and mitigating malnutrition and side effects through proper nutritional and physical well-being monitoring. We aim to deliver this digital nutrition service via the Nootric app, thereby promoting patients’ adherence to a healthy lifestyle.

In this study, we included under the term “oncohematological patient” those who met the inclusion criteria: adults over 18 years of age with a diagnosis of oncohematologic pathology undergoing active treatment. The hematologic malignancies included were mostly lymphoproliferative syndromes and multiple myeloma. We analyzed only nutritional and physical parameters within this group.

### Objectives

We evaluated an intervention designed to support oncohematological patients in active treatment. The primary goal was to assess how eHealth tools in nutrition and well-being management impact patients in oncohematology. Additionally, we aimed to determine the usefulness of physical-nutritional management in improving tolerance to chemotherapy treatments within routine clinical practice.

As secondary objectives, on the one hand, we aimed to evaluate the usefulness of the application of a physical-nutritional intervention among oncohematological patients by observing serial cases. On the other hand, we wanted to evaluate the adequacy and acceptability of this app in this group of patients. We aimed to qualitatively evaluate how knowledge guides the reorientation of intervention strategies regarding physical-nutritional well-being in these patients. Finally, we aimed to understand the nutritional and physical requirements throughout each phase of these patients' treatment, considering the potential implications for their well-being. Measures of engagement with the intervention and semistructured interviews with intervention participants were used to evaluate the feasibility of the intervention.

## Methods

### Study Design

**Figure 1:**
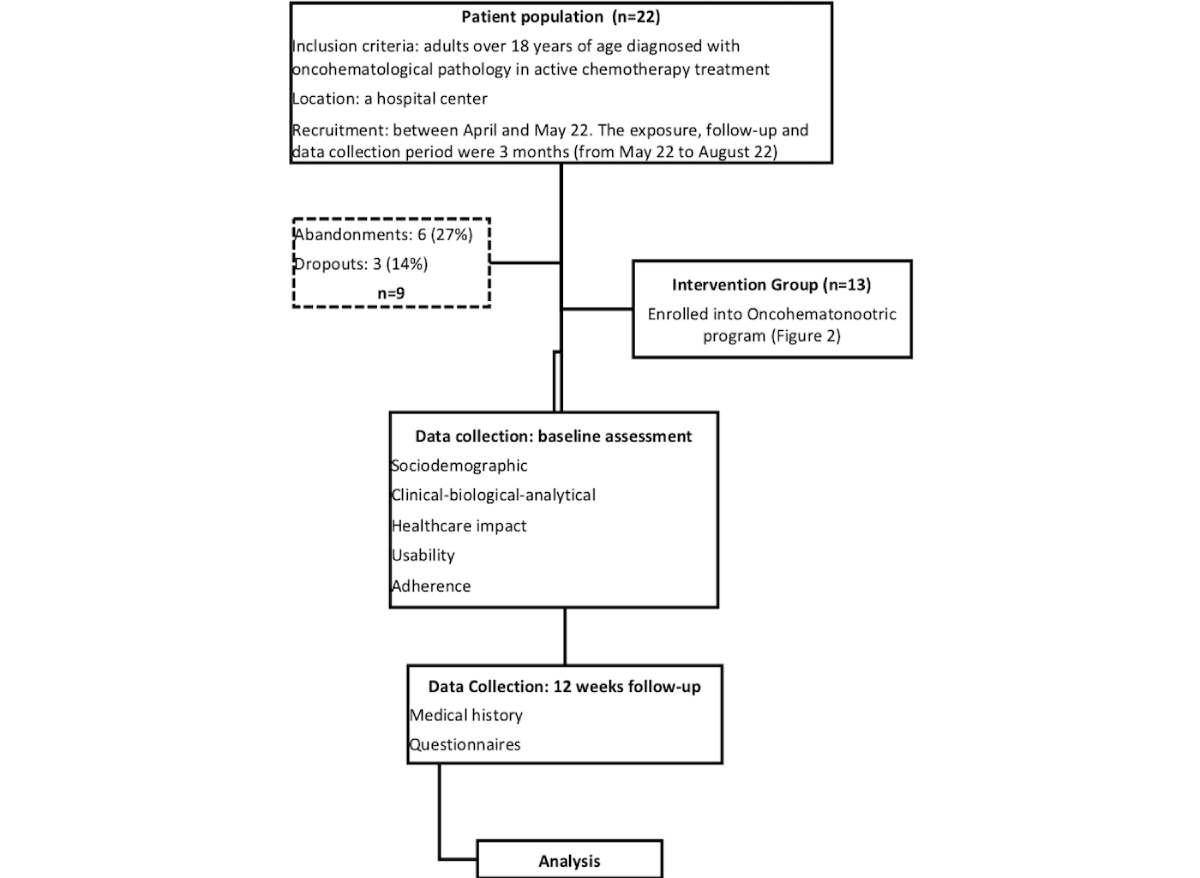
Study flow diagram.

A descriptive, observational, prospective, longitudinal cohort pilot study was conducted among oncohematological patients in our hospital center (Figure 1).

Recruitment took place between April and May 22. The exposure, follow-up, and data collection period lasted 3 months (from May 22 to August 22). The sample consisted of 22 patients and was not divided under any concept at the beginning of the study. During study development, patients who had good adherence were included in the exposed cohort. However, those who dropped out of the study and had a low adherence rate were included in the unexposed cohort. They were not taken into account in the analysis of the results, and no comparative study was performed. To ensure that older adults were not excluded due to the digital gap, we included patients aged above 80 years old, and during the first visit, we encouraged them to continue using the Nootric app.

### Study Setting

This study was conducted in the province of Alicante, Spain, at Vialopó University Hospital, which tends to a culturally, linguistically, and socioeconomically diverse population. The participants were recruited from the hospital’s hematology department.

### Participants

Patients were eligible to participate if they (1) were aged ≥18 years, (2) had a documented diagnosis of oncohematological pathology, and (3) were receiving medical treatment for cancer at the time of study initiation. Patients were excluded from the study if they (1) were children, adolescents, or pregnant women; (2) were due for surgery with hospital nutritional treatment; (3) were following nutritional care or hospital treatment; (4) had any acute or chronic condition that the practitioner believed limited their ability to participate in the study; (5) were unable to provide written consent; (6) were not literate; and (7) did not have a smartphone.

### Intervention: OncohematoNootric Program

#### Overview

In recent years, there has been a growing use of eHealth tools, such as mobile apps, in nutritional interventions with good acceptance and results [2,3]. These technologies have been applied in different health care settings, such as mental health support and chronic disease management, to enable interaction with patients and promote engagement with health care interventions. Ultimately, they aim to increase the acceptability, use, and effectiveness of interventions [17,18]. However, to date, few studies have evaluated the effectiveness of these apps in routine clinical practice within the health care environment. There have been no studies in patients with hematological malignancies undergoing treatment [4].

The OncohematoNootric program involves a continuous approach and follow-up of patients by their physician and nutritionist through the Nootric app over 3 months via face-to-face/telematic consultations, voice calls, and direct chat through the app.

The intervention aims to provide nutritional-physical support tools to oncohematology patients receiving treatment who may develop adverse effects that put their physical and nutritional well-being at risk. The program provides training and information. It also facilitates risk assessment and clinical support, as needed, with the help of a physician-nutritionist alarm system.

#### Education Website

Nootric is a digital nutrition service that creates personalized nutrition plans tailored to the oncohematological patient, featuring recipes compatible with the potential side effects of treatment. It augments cognitive-behavioral therapy and provides guides and challenges that address aspects related to nutrition and its application in daily life. Physicians can monitor patients through the app using a dynamic panel that displays real-time actions. It also includes a chat feature to communicate with a dietitian-nutritionist.

In this study, all patients received dietary and exercise recommendations from professionals. The Nootric app aimed to help patients improve their health and well-being by facilitating behavioral changes.

#### Intervention Development and Patient Involvement

The intervention was designed in conjunction with patients, medical and nutritional health professionals, and professionals with expertise in eHealth and wellness management programs and technologists. No generative artificial intelligence (AI) was used in this study.

For program implementation, a training session on the use of the Nootric app was held with different medical teams. When the candidate patient was selected by the center, the team gave them a patient information sheet, an informed consent form, and an information leaflet. Once the patient agreed to participate in the pilot, they handed over the signed informed consent form and began to participate. The center registered the patient on the Nootric website by entering a code assigned to the patient. Once this registration was completed, the patient downloaded the app and logged in. Next, the patient completed a series of forms that served as a basis for the dietitian-nutritionist to establish their personalized plan. In addition, a clinical-biological test was performed via a blood test requested by the medical team and carried out at the hospital, after which the results were recorded, and the nutritional counseling intervention was initiated. At the beginning of the intervention, the health care professionals oriented the patient on the use of the Nootric application and emphasized the importance of good nutrition and physical exercise. Each patient received a weekly menu and shopping list, was able to upload photos of their meals during follow-up, and had direct access to an app-based chat with a nutritionist. During the study, the patient was able to contact their dietitian-nutritionist through the app’s chat function to solve nutritional doubts and receive motivational support to increase physical activities and food recording.

During the project, improvements were made to the app to provide better patient care, including adapting 70 menu prescriptions to be compatible with the potential side effects of the treatment, configuring menu items to ensure suitability for the most common side effects, and preparing and adapting informative guides and challenges. Other improvements included sending activity and hydration reminders, optimizing the internal messaging system for medical professionals to exchange information with nutritionists, and making functional changes to facilitate uploading files for medical professionals.

An action plan was created to generate alerts between the physician and nutritionist with all the possible adverse events that patients could present (eg, hyporexia, weight loss, skin and nail changes, diarrhea, dyspepsia, pain according to a visual analog scale, edema, fatigue, constipation, dysphagia, odynophagia, mucositis, canker sores, nausea, vomiting, diarrhea, insomnia, urinary and bladder problems, anuria, bleeding, flu-like symptoms, fever, xerostomia, rash, and pruritus), along with their severity criteria and the action plan to be followed by the doctor and nutritionist.

When any of these issues were detected, the nutritionist informed the doctor, who carried out an unscheduled telematic/presential consultation with the patient to resolve the issue with the nutritionist’s support. On the other hand, if the issue was detected by the doctor, the latter informed the nutritionist so that the patient could receive support from both. In this way, both professionals were always kept up to date on incidents and procedures. The communication channel between professionals was the Nootric web platform.

For patients who were observed to have low adherence to the program during the pilot, the professionals studied the potential causes and intensified their actions to avoid dropout.

During the follow-up period, a weekly evaluation of the variables under study was carried out by the professionals. Biweekly follow-up meetings were held with the team to track the program and make possible improvements. Patients completed weekly program evaluation forms. After the follow-up study, a satisfaction survey was conducted to qualitatively evaluate patient satisfaction and program usefulness.

**Figure 2:**
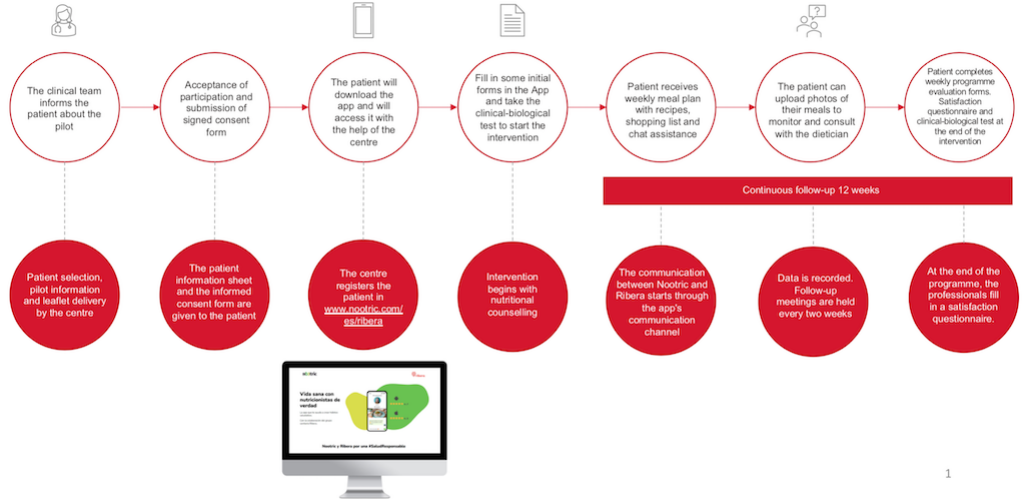
Flowchart of the patients with the medical professionals and nutritionists during the study.

A new clinical-biological test was performed to comparatively analyze the results obtained before and after the intervention (Figure 2).

### Study Outcomes

The primary outcome of the study was the health impact of the use of an eHealth tool related to nutrition and physical well-being on the oncohematological patient.This was measured by determining the following variables: nº alerts resolved, nº emergency visits, nº unscheduled consultations, nº treatments suspended, nº hospital admissions and improvement in nutritional habits according to the patient's perception, nº patients referred to the hospital’s Nutrition Unit, nº patients requiring adjustment of support treatment, and nº patients with gastrointestinal toxicity (which determines the impact on improving tolerance to treatments). The secondary outcomes included assessing the perceived improvement in the patients’ physical and nutritional well-being, determined through satisfaction and usefulness questionnaires at the end of the intervention. We also sought to assess the feasibility of the intervention, focusing on usability, acceptability, and adherence regarding different intervention components.

### Data Collection and Study Procedures

The variables were collected on a form based on Microsoft Office Excel 2021 (Microsoft Corp), with each coded and subsequently exported to the SPSS statistical software (version 28.0; IBM Corp). To describe continuous variables with normal distribution, measures of central tendency were used, such as arithmetic mean. The qualitative variables were presented as frequency and proportion.

The variables collected at the beginning of the pilot study, during follow-up, and at the end were: (1) sociodemographic, including sex, age, level of education, main disease, comorbidities, and medication; (2) clinical-biological-analytical, with the following parameters included in the blood analysis to assess nutritional status and treatment toxicity and direct the professionals’ action plan: hemoglobin, creatinine clearance, calcium, total protein, albumin, vitamin B12, folic acid, ferritin, malnutrition index, total cholesterol, liver enzymes aspartate aminotransferase, alanine aminotransferase, alkaline phosphatase, and gamma-glutamyl transferase, cancer medical treatment scheme, food consumption patterns measured through the form provided by Nootric through the app, and BMI; (3) health care impact; (4) usability; and (5) adherence.

To weight the usability of the application, the following user interaction variables were considered: viewing, rating, comments on recipes, uploaded photos, access to the chat, review of a guide or completion of a challenge (framed in cognitive-behavioral training), points achieved (result of the gamification provided by Nootric), and exercises displayed. Adherence was determined by analyzing each patient’s use of the Nootric app during the program application period. For adherence, the percentage of access to the Nootric application was calculated, considering the 120-day access as 100% adherence.

**Figure 3:**
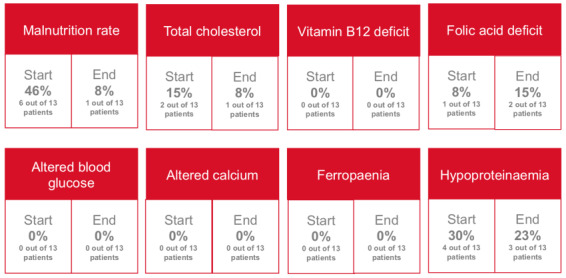
Biological test: Relevant data that were observed at baseline and the end of the study (n=13).

**Figure 4:**
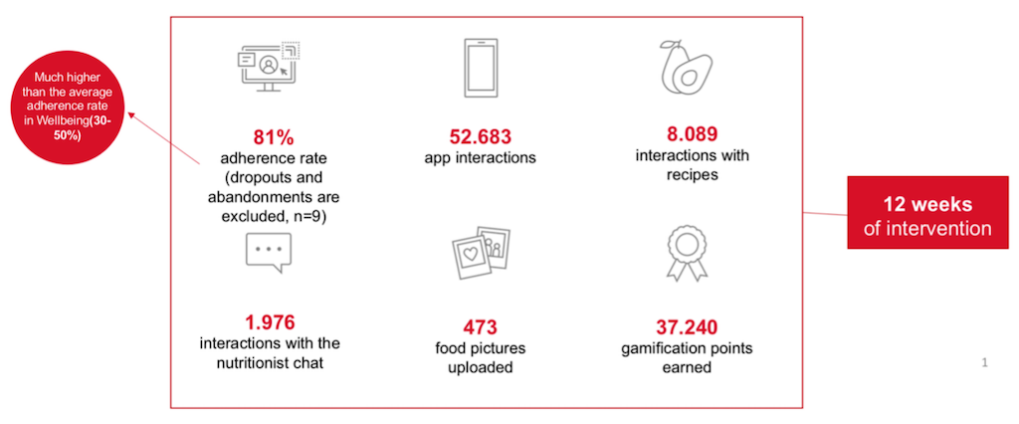
Results regarding usability data, user experience, and adherence rate: A high level of interaction was achieved with patients using the service provided by the nutrition and medical team via the app.

**Figure 5:**
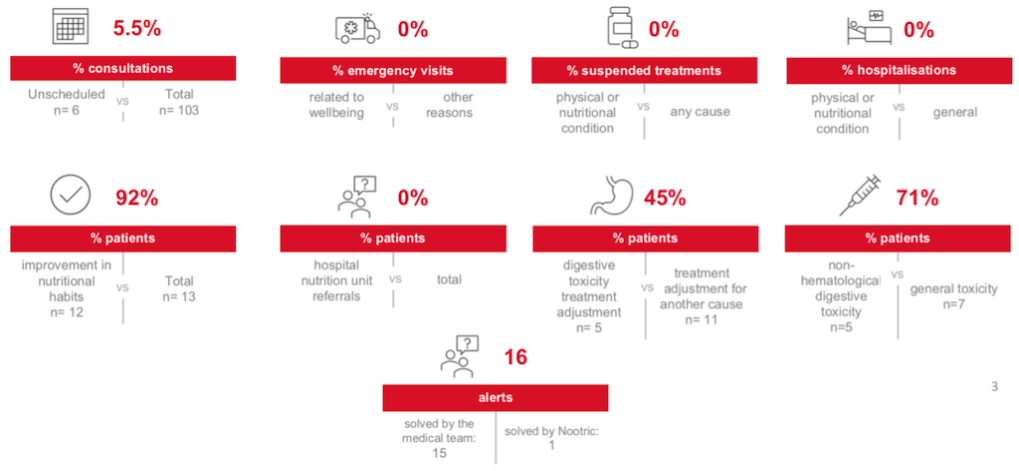
Results of the health care impact: Attendance data and rates.

The evaluations performed can be viewed in more detail in Tables 1 and 2 and Figures 3-5. The data extracted from the patients' medical records included results of the blood analysis, main disease, comorbidities and their medication, cancer medical treatment scheme, and health care impact variables.

**Table 1:** Variables related to patients' sociodemographic characteristics, oncohematological diseases, cancer medical treatment received during the study, and adherence to the program during 12 weeks of intervention (N=22).

Variable and description		Patients and program adherence (N=22), n (%)
**Socioeconomic level**		
	No formal education	8 (36)
	Primary education	7 (32)
	Vocational education	4 (18)
	Secondary education	2 (9)
	Higher education	1 (5)
**Comorbidity**		
	Hypertension	13 (59)
	Dyslipidemia	8 (36)
	Arthritis	17 (77)
	Diabetes mellitus	3 (14)
**Oncohematological diseases^a^**		
	Lymphoproliferative syndromes	13 (59)
	Multiple myeloma	4 (18)
	Myelodysplastic syndrome	1 (5)
	Chronic myeloid leukemia	4 (18)
**Cancer-directed treatment**		
	Immunochemotherapy^b^	15 (68)
	Targeted biological or targeted therapy^c^	7 (32)
**Program adherence**		
	Total abandonments^d^	6 (27)
	Dropouts (adherence <7%)	3 (14)

^a^Patients received medical treatment, physical-nutritional recommendations, and close monitoring by their multidisciplinary assistance program. Most of the study population consisted of patients without severe comorbidities (18/22, 80%).

^b^These include rituximab, cyclophosphamide, doxorubicin, vincristine, and prednisone, (R-CHOP); adriamycin, bleomycin, vinblastine, and dacarbazine (ABVD); rituximab, bortezomib, cyclophosphamide, adriamycin, and prednisone (VR-CAP); and methotrexate, regimens with melphalan, and 5-azacitidine.

^c^Therapeutic regimens included daratumumab, bortezomib, venetoclax, obinutuzumab, ibrutinib, imatinib, and nilotinib.

^d^These included 3 women and 3 men. Dropouts were considered as users with adherence rates below 7%, and they were not accounted for in the analytical results, body variables, and interactions with the app.

**Table 2:** Clinical-biological-analytical laboratory assessment.

Laboratory parameters analyzed at the beginning and end of the study	Patients who had abnormalities at the beginning of the study (n=13), n (%)	Patients who had abnormalities at the end of the study (n=13), n (%)
Altered renal function	0 (0)	0 (0)
Altered blood glucose	0 (0)	0 (0)
Altered calcium	0 (0)	0 (0)
Elevated total cholesterol	2 (15)	1 (8)
Altered transaminases	1 (8)	1 (8)
Iron deficiency	0 (0)	0 (0)
Elevated malnutrition index	5 (38)	1 (8)
Hypoproteinemia	4 (30)	3 (23)
Vitamin B12 deficiency	0 (0)	0 (0)
Folic acid deficiency	1 (8)	2 (15)
Anemia	3 (23)	3 (23)

### Ethical Considerations

This study was approved by the Ethics Committee of Vinalopó University Hospital on March 30, 2022. All research activities involving human patients in this study have been treated in accordance with the ethical guidelines established by The Organic Law on Data Protection. All necessary approvals were obtained, including for the analysis of the research data.

This study complies with the ethical provisions outlined in the informed consent, and any additional analysis has been conducted in accordance with the existing ethical approvals. Informed consent was obtained from all patients for the conduct of this study and publication of this article, and no compensation was provided. Patients were informed that the doctor would receive information about their progress.

To protect the privacy and confidentiality of the participants, all data collected in this study were anonymized before the analysis. Measures were taken to ensure that the participants’ identifiable details were not disclosed. We used an anonymous identification system that consisted of assigning an alphanumeric code to each patient registered on the Nootric web platform and in the Nootric app. No one else, apart from Ribera's medical team and Nootric's team of nutritionists, had access to the data in the Nootric app and website, which are confidential and encrypted.

The original informed consent allows for secondary analysis without additional consent; this includes data collected from the participants’ medical history.

## Results

### Sociodemographic Variables

Of the 22 included patients, 11 (50%) were female and 11 (50%) were male, with an average age of 70 (range 42-84) years. Among them, 13 (60%) patients were under 65 years and 9 (40%) were over 65 years. The variables regarding sociodemographic characteristics, treatment received during the study, and program adherence are described in Table 1.

### Clinical-Biological-Analytical Variables

A clinical-biological test was conducted for all patients at the beginning of the study, but only 13 (59%) out of 22 patients completed it. On this population, we performed the analysis of the results. Table 2 presents all the analyzed laboratory parameters and the results at the beginning and end of the

intervention. Some of the relevant data are shown in Figure 3. Some patients received corticosteroid therapy, which increases the risk of developing steroidal hyperglycemia. These patients benefited from an adapted physical-nutritional plan. There were no patients in the study who presented with alterations in blood glucose levels. Of the 2 (15%) patients who had dyslipidemia at baseline, 1 (8%) patient did not maintain dyslipidemia at the end of the study. Furthermore, 1 (8%) patient presented with iron overload secondary to a high transfusion requirement. Iron chelation therapy was initiated, which triggered a grade 3 hepatotoxicity. After discontinuing it, it improved clinically and analytically to grade 1. Of 6 (46%) patients with malnutrition at baseline, only 1 (8%) patient still had malnutrition at the end of the study. Of the 4 (30%) patients who presented with hypoproteinemia at baseline, 1 (23%) did not have it at the end. Moreover, 1 patient (8%) presented with a folic deficit at baseline and maintained it at baseline despite having received supportive treatment and dietary recommendations.

The clinical interview confirmed that treatment compliance was inadequate. Among 13 patients who had anemia at the beginning of the study, 3 (23%) did not maintain it at the end of the study. Conversely, 3 (23%) of the 13 patients who had anemia at the end of the study did not have it at baseline. None of the causes were caused by vitamin B12, folic acid, or iron deficiency but by myelotoxicity due to targeted cancer therapy.

### Program Adherence and Usability

A total of 6 (27%) out of 22 patients abandoned the study due to a lack of adherence to the program, attributed to advanced age, insufficient socioeconomic level to ensure proper use of the app, lack of family support for improving adherence, and lack of initiative to establish a change in the physical-nutritional routine. They were not taken into account in the analysis of the results. In addition, users with less than 7% adherence were considered dropouts (3/22, 14%), and they were not taken into account in the analysis of the results. None of these patients were older adults or dropped out due to the digital gap.

Excluding dropouts and abandonments (n=9, 30%), the adherence rate was 81%, established by calculating the arithmetic mean of the adherence rates of 13 patients, much higher than the average rate in well-being. Of the 22 patients, between 7 and 11 (30%-50%) perceived an improvement in well-being, determined by the satisfaction survey conducted at the end of the study. To determine the adherence rate of each patient, the use of the app by the patients was evaluated and the parameters were nº interactions with the app, use of the chat with the nutritionist, nº interactions with the recipes, nº photos of recipes uploaded by the patients, and gamification points earned (Figure 4). Regarding the impact of usability, we obtained an average of 655 impacts per app user (Figure 4).

### Impact on Health Care

Regarding the impact on health care quality, Table 2 and Figure 5 show data that demonstrates highly satisfactory results. None of the total emergency visits were related to physical and nutritional well-being.

No hospitalizations occurred during the study for any cause, including those related to physical and nutritional well-being. No patient had to be referred to the hospital’s Nutrition Unit. Of the total number of medical consultations carried out, only 5.5% (6/103) were unscheduled and none of them were carried out for physical or nutritional issues. A total of 7 patients presented toxicity, among which 5 (71%) were cases of digestive toxicity. Of the 11 patients who required adjustment of supportive or symptomatic treatment due to toxicity, 5 (45%) had digestive toxicity. No treatment was suspended due to physical or nutritional conditions.

The patients showed an improvement in tolerance to chemotherapy treatments since there were no cases of grade 3 or higher gastrointestinal toxicity, defined as complications requiring intravenous support treatment. There were also no hospitalizations, emergency visits, or chemotherapy treatment discontinuations for this reason. A high percentage of patients (12/13, 92%) perceived an improvement in their nutritional habits.

### Impact on Patients’ Perceived Improvements in Physical and Nutritional Well-Being

At the end of the study, the Nootric team disseminated an anonymous survey to measure satisfaction and usefulness for the patients and medical professionals involved. This enabled us to evaluate the impact on the perceived improvement in the patients’ physical and nutritional well-being. We observed that the users who adhered adequately to the program showed an improvement in this aspect. A total of 12 questionnaires were filled out by patients.

The average satisfaction rating among professionals was 4.8 out of 5, while patients rated their satisfaction at 4.3 out of 5. Table 3 highlights these results.

**Table 3:** Results of the impact on the perceived improvement in patients’ physical and nutritional well-being. A satisfaction and usefulness questionnaire was carried out for the patients involved at the end of the study (n=13).

Question	Score out of 5
To what extent do you feel that the nutritional wellness program has enabled you to improve your eating habits?	4
Do you think your illness has allowed you to use the program correctly?	4.08
To what degree would you like to continue using this tool as part of your day-to-day life beyond the pilot study?	
What is the degree of satisfaction with the nutritional wellness program?	4.25
What is the degree of satisfaction with the nutritional professionals who have assisted you in the program?	4.92
Do you think the app is easy to use?	4.42

## Discussion

### Context

Our study is based on a multidisciplinary nutritional and exercise support program for oncohematological patients undergoing active treatment using new technologies. This study provides initial data on the effectiveness of a novel physical and nutritional support program aimed at patients with malignant hemopathies (largely represented in our study as lymphoproliferative syndromes and multiple myeloma) receiving targeted cancer treatment. It also provides a detailed evaluation of the implementation, adoption, and overall acceptability of this digital care intervention through a mobile app. The evaluation design has been adapted to the study objectives to provide new data to enable a better estimation of such an intervention’s impact and inform further development of digital care interventions for malignant blood diseases under active treatment.

### Principal Findings

We did not observe any hospital admissions or discontinuation of chemotherapy treatment related to the patients' physical-nutritional well-being, supporting the benefit of the program. Adequate nutritional support was provided to ensure patients' well-being and mitigate the need for referral to the hospital's Nutrition Unit. Regarding the impact on physical and nutritional well-being, we observed that users who adhered adequately to the program improved in this aspect.

We observed a reduction in the number of unplanned consultations related to physical and nutritional well-being. In terms of impact on health care quality, the results demonstrated highly satisfactory results. None of the total emergency visits were related to physical and nutritional well-being. Moreover, the intervention received a high satisfaction rating from both professionals and patients.

Regarding other works in the field, there is little information about the best nutritional support for patients with cancer [9,19,20]. Antineoplastic agents are known to be associated with gastrointestinal complications that lead to physical and nutritional repercussions, which can decrease well-being and result in death due to malnutrition [19]. Additionally, early nutritional intervention can improve prognosis and reduce the disease’s complication rate [12,19].

One study used a novel mobile app to assess and evaluate dietary behaviors in 39 oncologic patients. Although 5 patients dropped out prior to the study, the authors concluded that participants who tracked their daily dietary habits using a mobile phone app were more likely to reach their nutritional goals than the control patients. Other studies have used mobile apps to record nutritional status and activity levels in patients with breast cancer or other or other diseases, but none of them are similar to our study [20]. Our study was performed by a multidisciplinary team using both the app and the internet to maintain contact with the patients. Furthermore, the multidisciplinary team tailored each patient's diet to suit their individual needs throughout their cancer treatment journey, particularly addressing gastrointestinal toxicities associated with active chemotherapy. This underscores the effectiveness of such technologies for integration into clinical practice without compromising the human touch in health care delivery.

### Limitations and Strengths

This study highlights the importance of eHealth programs in addressing nutrition and

well-being among oncohematology patients, offering significant value in multidisciplinary care management. The use of the Nootric app allowed for improved health care indicators and physical-nutritional well-being, promoting better patient outcomes.

Another notable strength of this study is the finding that over 50% (n=11) of the patients improved their physical-nutritional habits, leading to a considerable enhancement in their perception of well-being.

In terms of limitations, we must point out that this study has a small sample size of 22 patients, which may limit the generalizability of the results. Moreover, we experienced a 27% (6/22) dropout rate due to a lack of adherence to the program, which could have affected the overall results. In addition, not all of those who completed the study completed the clinical-biological tests. Finally, we acknowledge that the 12-week follow-up period might not adequately capture the program’s clinical impact on adherence to healthy habits and improved physical and nutritional well-being.

### Conclusion

In conclusion, using targeted eHealth programs for nutrition and well-being among oncohematological patients undergoing active treatment offers significant value in multidisciplinary care management. This is achieved through enhanced interaction between physicians, dietitian-nutritionists, and patients via a digital nutrition service, such as the Nootric app. Supporting patients throughout their cancer journey, these technologies serve as valuable tools for integration into clinical practice without detracting from the human aspect of health care. Therefore, implementing projects that leverage new technologies in routine holistic clinical practice for oncohematological patients could prove cost-effective in both the short and long term. By facilitating the early detection of health issues related to physical-nutritional well-being and anticipating potential complications, these initiatives may help reduce unscheduled visits and admissions related to this condition.

## Abbreviations

AI: artificial intelligence
ICT: information and communication technology
VAS: visual analog scale

## Data Availability

Our data adheres to open science availability guidelines for broader research accessibility.
